# Supplementary material for: Endogenous Real Time Imaging Reveals Dynamic Chromosomal Mobility During Ligand-Mediated Transcriptional Burst Events
Source: bioRxiv. 2025 Aug 19:2025.08.18.670875. Preprint. [Version 1] doi: 10.1101/2025.08.18.670875 (PMC12393356; doi:10.1101/2025.08.18.670875)
Supplement: Supplement 1 [file NIHPP2025.08.18.670875v1-supplement-1.pdf]

bioRxiv preprint doi: https://doi.org/10.1101/2025.08.18.670875; this version posted August 19, 2025. The copyright holder for this preprint (which was not certified by peer review) is the author/funder, who has granted bioRxiv a license to display the preprint in perpetuity. It is made available under aCC-BY 4.0 International license.

Supplemental Figure 1. DNA visualization of *NRIP1* via CymR protein fused to either mTurquoise2, mKate, or Halo JaneliaFluor-549.

bioRxiv preprint doi: https://doi.org/10.1101/2025.08.18.670875; this version posted August 19, 2025. The copyright holder for this preprint (which was not certified by peer review) is the author/funder, who has granted bioRxiv a license to display the preprint in perpetuity. It is made available under aCC-BY 4.0 International license.

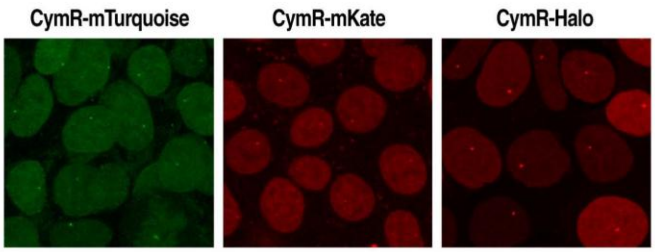

598

bioRxiv preprint doi: https://doi.org/10.1101/2025.08.18.670875; this version posted August 19, 2025. The copyright holder for this preprint (which was not certified by peer review) is the author/funder, who has granted bioRxiv a license to display the preprint in perpetuity. It is made available under aCC-BY 4.0 International license.

Supplemental Figure 2. ERα SMT. **a.** Agglomerative clustering identified 6 distinct ERα populations characterized by alpha and diffusion in minus E2, acute, and chronic states. MSD curves of a randomized subset of single molecules from each cluster are graphed. **b.** GMM clustering similarly identified 6 distinct ERα populations characterized by alpha and diffusion in minus E2, acute, and chronic states. MSD curves of a randomized subset of single molecules from each cluster are graphed.

bioRxiv preprint doi: https://doi.org/10.1101/2025.08.18.670875; this version posted August 19, 2025. The copyright holder for this preprint (which was not certified by peer review) is the author/funder, who has granted bioRxiv a license to display the preprint in perpetuity. It is made available under aCC-BY 4.0 International license.

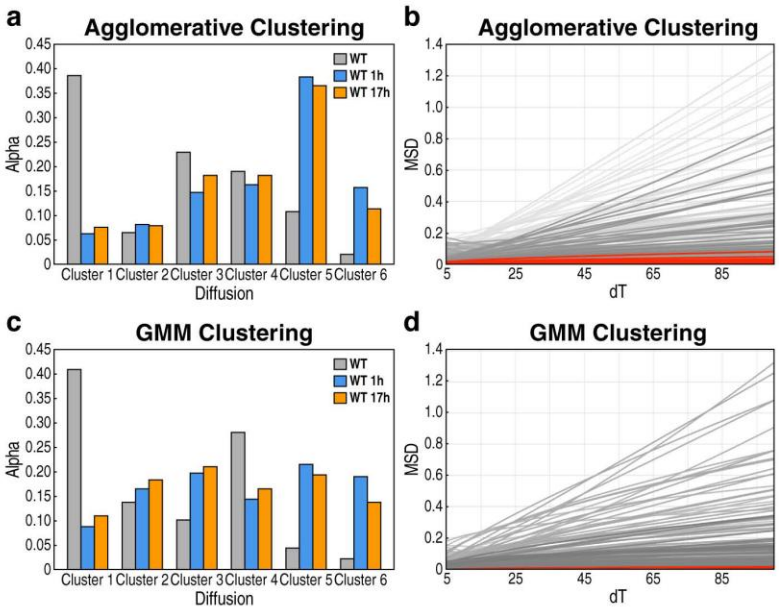

bioRxiv preprint doi: https://doi.org/10.1101/2025.08.18.670875; this version posted August 19, 2025. The copyright holder for this preprint (which was not certified by peer review) is the author/funder, who has granted bioRxiv a license to display the preprint in perpetuity. It is made available under aCC-BY 4.0 International license.

Supplemental Figure 3. TFF1 FISH. **a.** Immuno-DNA FISH of *TFF1* shows increased proximity to SC35 in acute phase relative to chronic. **b.** Immuno-DNA FISH of *TFF1* shows induced association with Matrin3 network in the chronic state relative to acute. **c.** Immuno-RNA FISH of *TFF1* shows increased proximity to SC35 in acute relative to chronic phase.

bioRxiv preprint doi: https://doi.org/10.1101/2025.08.18.670875; this version posted August 19, 2025. The copyright holder for this preprint (which was not certified by peer review) is the author/funder, who has granted bioRxiv a license to display the preprint in perpetuity. It is made available under aCC-BY 4.0 International license.

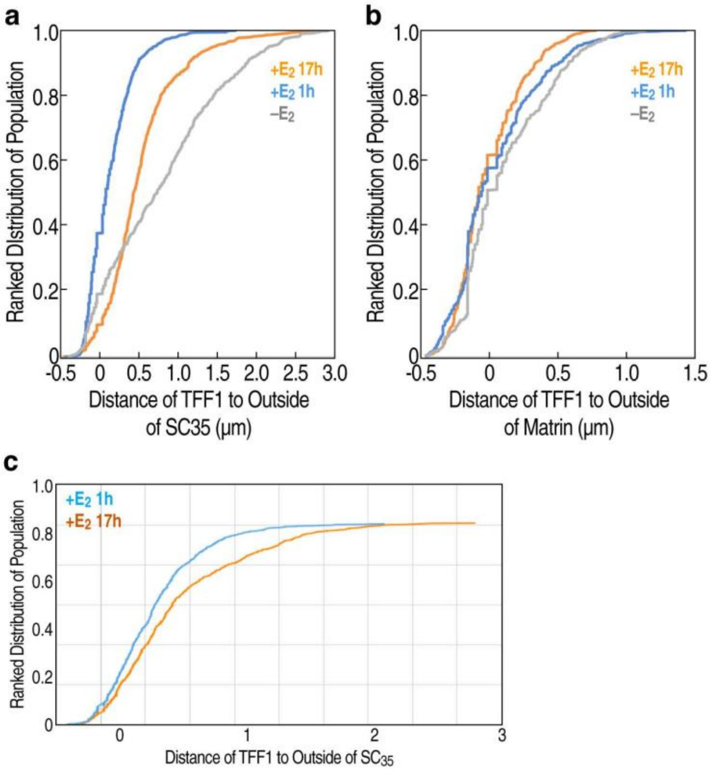

610

bioRxiv preprint doi: https://doi.org/10.1101/2025.08.18.670875; this version posted August 19, 2025. The copyright holder for this preprint (which was not certified by peer review) is the author/funder, who has granted bioRxiv a license to display the preprint in perpetuity. It is made available under aCC-BY 4.0 International license.

Supplemental Movie 1. *NRIP1* RNA visualization timelapse of burst events.

Supplemental Movie 2. *NRIP1* DNA visualization timelapse of mobility.

Supplemental Movie 3. Single molecule timelapse of ERα in minus E_2 condition.

Supplemental Movie 4. Single molecule timelapse of ERα in acute E_2 condition.

Supplemental Movie 5. Single molecule timelapse of ERα in chronic E_2 condition.

Supplemental Movie 6. Segmented timelapse of *NRIP1* RNA, DNA, and interchromatin granule 3-color visualization.
